# SDF-1/CXCR4 Signaling Preserves Microvascular Integrity and Renal Function in Chronic Kidney Disease

**DOI:** 10.1371/journal.pone.0092227

**Published:** 2014-03-17

**Authors:** Li-Hao Chen, Suzanne L. Advani, Kerri Thai, M. Golam Kabir, Manish M. Sood, Ian W. Gibson, Darren A. Yuen, Kim A. Connelly, Philip A. Marsden, Darren J. Kelly, Richard E. Gilbert, Andrew Advani

**Affiliations:** 1 Keenan Research Centre for Biomedical Science and Li Ka Shing Knowledge Institute of St. Michael's Hospital, Toronto, Ontario, Canada; 2 Ottawa Hospital Research Institute, University of Ottawa, Ottawa, Ontario, Canada; 3 Health Sciences Centre, University of Manitoba, Winnipeg, Manitoba, Canada; 4 Department of Medicine, St. Vincent's Hospital, Melbourne, Victoria, Australia; Universidade de Sao Paulo, Brazil

## Abstract

The progressive decline of renal function in chronic kidney disease (CKD) is characterized by both disruption of the microvascular architecture and the accumulation of fibrotic matrix. One angiogenic pathway recently identified as playing an essential role in renal vascular development is the stromal cell-derived factor-1α (SDF-1)/CXCR4 pathway. Because similar developmental processes may be recapitulated in the disease setting, we hypothesized that the SDF-1/CXCR4 system would regulate microvascular health in CKD. Expression of CXCR4 was observed to be increased in the kidneys of subtotally nephrectomized (SNx) rats and in biopsies from patients with secondary focal segmental glomerulosclerosis (FSGS), a rodent model and human correlate both characterized by aberration of the renal microvessels. A reno-protective role for local SDF-1/CXCR4 signaling was indicated by i) CXCR4-dependent glomerular eNOS activation following acute SDF-1 administration; and ii) acceleration of renal function decline, capillary loss and fibrosis in SNx rats treated with chronic CXCR4 blockade. In contrast to the upregulation of CXCR4, SDF-1 transcript levels were decreased in SNx rat kidneys as well as in renal fibroblasts exposed to the pro-fibrotic cytokine transforming growth factor β (TGF-β), the latter effect being attenuated by histone deacetylase inhibition. Increased renal SDF-1 expression was, however, observed following the treatment of SNx rats with the ACE inhibitor, perindopril. Collectively, these observations indicate that local SDF-1/CXCR4 signaling functions to preserve microvascular integrity and prevent renal fibrosis. Augmentation of this pathway, either purposefully or serendipitously with either novel or existing therapies, may attenuate renal decline in CKD.

## Introduction

The progressive decline of renal function in chronic kidney disease (CKD) is characterized by both fibrotic scarring of the kidney and obliteration of the renal microvessels, these two pathogenetic hallmarks commonly occurring in tandem and enjoying a reciprocal relationship. On the one hand, microvascular loss may occur as a result of the occlusive actions of accumulating matrix proteins, whereas on the other hand the same process may itself contribute to organ fibrosis and progressive renal decline by predisposing the kidney to hypoxic injury [1]. Coupled with an increasing appreciation for the pivotal role that angiogenic factors may play in renal development [2], homeostasis [3] and disease [4], [5], preservation of the glomerular and peritubular capillary architecture is thus a desirable characteristic of both existing and novel renoprotective therapies [6].

One pathway that has recently emerged as playing an essential role in renal vascular development is the stromal cell-derived factor-1α (SDF-1)/CXCR4 pathway [7]. SDF-1 is a CXC chemokine and the principal ligand for its cognate receptor, CXCR4, a seven transmembrane domain G-protein coupled receptor and the most prevalent chemokine receptor found in endothelial cells [8]. While originally defined for its role in maintenance of the hematopoietic stem cell niche and B-cell lymphopoiesis [9], the near ubiquitous tissue distribution of SDF-1 and its rapid degradation in blood indicate the capacity for much broader intra-organ specific functions [10]. The fundamental nature of the SDF-1/CXCR4 relationship is attested to by the development of identical defects in vasculogenesis and organogenesis that occur in the absence of either gene [11]. However, as with other angiogenic pathways [4], [5], [12], the role that SDF-1/CXCR4 signaling may play in the adult kidney appears to be context-dependent. For instance, studies in acute kidney injury support a reno-protective function for SDF-1/CXCR4 [13], whereas CXCR4-mediated hyperproliferation may actually contribute to the development of certain glomerular diseases [14].

Although reactivation of ontogenetic pathways is a common response of cells, tissues and organisms to a variety of injurious insults [15], the function of the developmentally essential SDF-1/CXCR4 pathway in CKD is unclear. Accordingly, in the present study we sought to combine studies conducted in experimental animals, cultured cells and human biopsy tissue to define the role of SDF-1/CXCR4 signaling in CKD, focusing on the bidirectional relationship between renal fibrosis and microvascular loss.

## Materials and Methods

### Ethics statement

Human biopsy studies were approved by the Institutional Research Board of the Health Sciences Centre, University of Manitoba. All patients gave written informed consent and the study was performed in accordance with the Declaration of Helsinki. All animal work was conducted according to the Canadian Council on Animal Care Guidelines. The specific experimental protocol, ACC 166, was approved by the Animal Care Committee of St. Michael's Hospital.

### Human studies

Localization of CXCR4 and SDF-1 was determined in kidney sections from patients who had undergone nephrectomy for tumor, with tissue removed from the opposite pole [16]. For gene expression studies, kidney tissue was obtained from patients with either secondary focal segmental glomerulosclerosis (FSGS) or time zero live kidney donors.

### Animals

#### Study 1

Expression of CXCR4 and SDF-1 was determined in the kidneys of sham (n = 6) and subtotally nephrectomized (SNx) (n = 8) rats after 8 weeks. Subtotal (5/6) nephrectomy or sham surgery was performed, in female Fischer 344 rats (F344, Charles River, Montreal, Quebec) aged 8 weeks, as previously described [17].

#### Study 2

For the study of chronic CXCR4 antagonism, female F344 rats aged 8 weeks underwent sham or subtotal nephrectomy surgery. Two days later, animals were randomized to receive either vehicle (PBS) or AMD3100 (1 mg/kg/day, Cayman Chemical, Ann Arbor, MI) s.c. and were followed for 8 weeks (sham, PBS n = 18, AMD3100 n = 12; SNx, PBS n = 14, AMD3100 n = 15). Glomerular filtration rate (GFR) was determined by FITC-inulin clearance [18]. Urine protein excretion was determined after 24 h metabolic caging. Systolic blood pressure (SBP) was measured with a 2F micro-manometer (Model SPR-838 Millar Instruments, Houston, TX) and analysed using Chart Software v5.6 (AD Instruments, NSW, Australia).

#### Study 3

In Study 3, we examined the effect of acute SDF-1 administration on glomerular signaling. Eight week old female F344 rats were first randomized to receive either PBS or AMD3100 (1 mg/kg) s.c. Four hours later, recombinant rat SDF-1 (10 μg/kg [19]; PeproTech, Rocky Hill, NJ) or vehicle (PBS) was delivered to the kidneys via the abdominal aorta (n = 6/group). To achieve this, the abdominal aorta was dissected, the right kidney was removed and the descending aorta was ligated distal and transiently ligated proximal to the renal artery. Either PBS or SDF-1 was delivered via an 18 G angiocath, circulation into the left kidney was then restored for 30 min before flushing the kidney with heparin (100 U), followed by 1 mL of PBS, with perfusion-exsanguination facilitated by severance of the external jugular vein. The kidney was then removed and glomeruli isolated by differential sieving [20].

#### Study 4

To determine whether either CXCR4 or SDF-1 mRNA were altered with ACE inhibition, real-time PCR was performed on mRNA isolated from the kidneys of sham (n = 8) and SNx (n = 7) Sprague Dawley rats after 12 weeks or SNx rats treated with the ACE inhibitor perindopril (8 mg/L in drinking water) (n = 8). The clinical characteristics of these rats have been previously described [21].

### Immunohistochemistry

Immunohistochemistry was performed as previously described [4], [16], [22] with antibodies in the following concentrations: SDF-1 1∶25 (R&D Systems, Minneapolis, MN), CXCR4 1∶50 (Abcam, Cambridge, MA), collagen IV 1∶100 (Southern Biotech, Birmingham, AL) and JG-12 1∶1000 (Bender Medsystems GbdH, Vienna, Austria). For quantitation of JG-12 and collagen IV immunostaining, kidney sections were scanned with the Aperio ScanScope system (Aperio Technologies Inc., Vista, CA) and analyzed using ImageScope (Aperio Technologies Inc.). Glomerular endothelial (JG-12) immunostaining was determined in 30 glomerular profiles from each rat kidney section [4], [23]. For estimation of peritubular JG-12 and tubulointerstitial collagen IV, the proportional area of positive immunostaining (excluding glomeruli) was determined in 10 randomly selected cortical fields (x100 magnification).

### Glomerulosclerosis Index

A minimum of 50 glomeruli were examined in PAS-stained kidney sections from each rat. The degree of sclerosis was subjectively graded on a scale of 0 to 4 as previously described [4].

### Fluorescent microangiography

Fluorescent microangiography (FMA) was performed in n≥3 rats/group as previously described [18]. Briefly, the abdominal aorta was ligated proximal to the renal artery and distally at the level of the aortic bifurcation and 1 ml of heparinized saline followed by 1 ml of 3% KCl were delivered, before perfusion with 100 ml 0.9% saline. A pre-warmed (40°C) agarose-fluorescent microbead mixture (1% low melting point agarose [Sigma] and 10% 0.02 μm fluospheres [Invitrogen, Carlsbad, CA]) was then delivered via an 18G angiocath. After infusion, the rat was cooled on ice and the kidney removed and fixed in 10% NBF. Subsequently, 200 μm thick kidney cross-sections were washed in PBS overnight and embedded in 95% 2,2′-thiodiethanol (Sigma). Serial images were collected with a confocal microscope (Leica TCS SL, Leica, Richmond Hill, ON) across the z-stack (0.8141 μm steps) in 6 glomeruli/rat. Glomerular capillary volume was calculated using ImageJ version 1.39 (National Institutes of Health, Bethesda, MD). Three dimensional reconstructions were generated using Neurolucida (MBF Bioscience, Williston, VT).

### Cell culture

*In vitro* experiments were conducted in NRK-49F renal fibroblasts (ATCC, Manassas, VA) and human umbilical vein endothelial cells (HUVECs) [4], [23]. NRK-49F cells were treated with 10 ng/ml recombinant rat transforming growth factor β (TGF-β) (R&D Systems) for 24 h, with or without pre-treatment with the histone deacetylase (HDAC) inhibitor vorinostat (5 μM) (Exclusive Chemistry, Obninsk, Russia) for 4 h. HUVECs were pre-incubated with 20 μM LY294002 (LC Laboratories, Woburn, MA), 1 μM AMD3100 or vehicle (0.1% DMSO) for 30 min before the addition of recombinant human SDF-1 (100 ng/ml) (R&D Systems) (or 1% BSA) for 30 min.

### Immunoblotting

Immunoblotting was performed as previously described [23] with antibodies in the following concentrations: phospho-eNOS Ser1177 1∶1000 (Cell Signaling, Danvers, MA), total eNOS 1∶2500 (BD Transduction Laboratories, Lexington, KY). Densitometry was performed using Image J.

### Real-time PCR

RNA was isolated from homogenized rat kidney tissue using TRIzol reagent (Life Technologies, Grand Island, NY). Total RNA (4 μg) was treated with RQ1 DNAse (1 U/μl) (Promega). For *in vitro* experiments, RNA isolation and DNase treatment of cultured cell extracts were performed using RNAspin Mini (GE Healthcare, Buckinghamshire, UK). DNase treated RNA (4 μg) was reverse-transcribed in a final volume of 25 μl using 0.5 μl AMV-RT (Roche Diagnostics, Laval, Quebec) in the manufacturer's buffer containing 1 mmol/L dNTPs, 0.5 μl RNase inhibitor (Roche) and 2 μg random hexamers (Amersham). Total RNA was extracted from human tissue using a Paradise Plus Reagent System (Arcturus, Mountain View, CA). SYBR green based real time PCR was performed on an ABI Prism 7900 HT Fast PCR System (Applied Biosystems, Foster City, CA) using the following primer sequences: rCXCR4, forward ATCATCTCCAAGCTGTCACACTCC, reverse GTGATGGAGATCCACTTGTGCAC; rSDF-1, forward GCTCTGCATCAGTGACGGTAAG, reverse TGGCGACATGGCTCTCAAA; rTGF-β, forward CACCCGCGTGCTAATGGT, reverse TGTGTGATGTCTTTGGTTTTGTCA; rRPL13a, forward GATGAACACCAACCCGTCTC, reverse CACCATCCGCTTTTTCTTGT; r18S, forward ATGTGGTGTTGAGGAAAGCAGAC, reverse GGATCTTGTATTGTCGTGGGTTCTG; hCXCR4, forward TGACGGACAAGTACAGGCTGC, reverse CCAGAAGGGAAGCGTGATGA; hSDF-1, forward AATTCTCAACACTCCAAACTGTGC, reverse TGCACACTTGTCTGTTGTTGTTC; hRPL32, forward CAACATTGGTTATGGAAGCAACA, reverse TGACGTTGTGGACCAGGAACT. Expression of the housekeeping genes did not differ between groups. Data analysis was performed using Applied Biosystems Comparative C_T_ method.

### Statistics

Data are expressed as means ± SEM except numerical proteinuria data which are presented as geometric mean ×/÷ tolerance factor. Statistical significance was determined by one-way ANOVA with a Newman-Keuls post-hoc comparison or Student's t-test where appropriate. Statistical analyses were performed using GraphPad Prism 5 for Mac OS X (GraphPad Software Inc., San Diego, CA).

## Results

### CXCR4 and SDF-1 localization in the adult kidney

**Figure 1:**
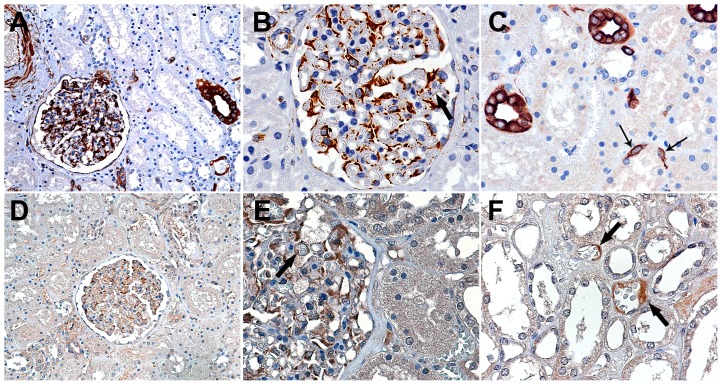
Immunostaining for SDF-1 (A–C) and CXCR4 (D–F) in adult human kidney. There is focal tubular (A and C) and glomerular podocyte staining for SDF-1. The thick arrows mark scattered positively staining glomerular and peritubular capillary endothelial cells; the thin arrows mark positively staining interstitial fibroblasts (C). (A and D) Original magnification x160. (B, C, E and F) Original magnification x400.

In our first experiments, we set out to define the sites of expression of SDF-1 and CXCR4 in the adult kidney. This initial immunostaining survey revealed constitutive expression of both SDF-1 and CXCR4 protein distributed prominently, although not exclusively, within the renal glomerulus (Figure 1). SDF-1 protein was notable within interstitial fibroblasts, podocytes, arteriolar smooth muscle and endothelial cells, epithelial cells of Bowman's capsule and scattered distal tubular cells, with weak, focal immunostaining within renal glomerular endothelial cells (Figure 1A-C). CXCR4 protein was present in glomerular podocytes and endothelial cells of the glomerular and peritubular capillaries (Figure 1D-F).

### Altered expression of SDF-1/CXCR4 in subtotally nephrectomized rats

**Figure 2:**
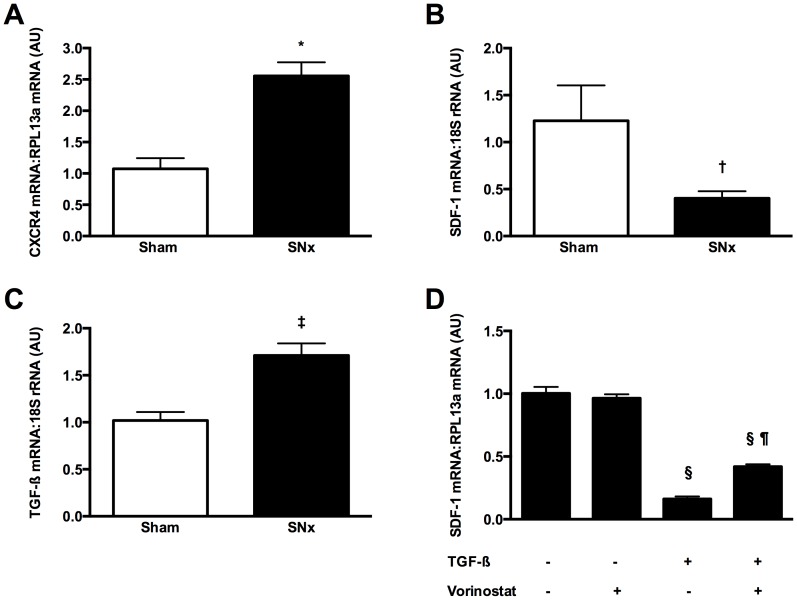
CXCR4 and SDF-1 mRNA in sham-operated and subtotally nephrectomized (SNx) rats. CXCR4 expression is increased in the kidneys of SNx rats (A), whereas SDF-1 is reduced (B). The kidneys of SNx rats also demonstrated an upregulation of the pro-fibrotic growth factor, transforming growth factor-β (TGF-β) (C). In cultured NRK-49F renal fibroblasts, recombinant TGF-β downregulated SDF-1 mRNA, with this effect being attenuated by HDAC inhibition with vorinostat (D). *p<0.001 vs. sham, ^†^p<0.05 vs. sham, ^‡^p<0.01 vs. sham, ^§^p<0.001 vs. control, ^¶^p<0.001 vs. TGF-β.

To elucidate the role of SDF-1/CXCR4 signaling in CKD we first determined gene expression of both receptor and ligand in the kidneys of rats that had undergone subtotal nephrectomy (SNx), a well-established model of progressive renal fibrosis. In these experiments, we observed an approximate two-fold increase in CXCR4 mRNA in the kidneys of SNx rats in comparison to sham-operated animals (Figure 2A). By way of contrast, SDF-1 mRNA was reduced in SNx kidneys (Figure 2B).

In exploring potential mechanisms that may mediate the downregulation of SDF-1 in SNx kidneys we considered the chemokine's prominent presence within interstitial fibroblasts and the sensitivity of these cells to the pro-fibrotic growth factor, TGF-β. TGF-β mRNA was increased >50% in the kidneys of SNx rats in comparison to sham animals (Figure 2C), whereas exposure of cultured NRK-49F renal fibroblasts to recombinant TGF-β resulted in an approximate 80% reduction in SDF-1 mRNA (Figure 2D). Consistent with an emerging recognition for the importance of post-translational protein acetylation in regulating the cellular response to TGF-β [24], SDF-1 downregulation was attenuated by pre-treatment of fibroblasts with the histone deacetylase (HDAC) inhibitor, vorinostat (Figure 2D).

### Chronic CXCR4 antagonism accelerates renal decline in subtotally nephrectomized rats

**Figure 3:**
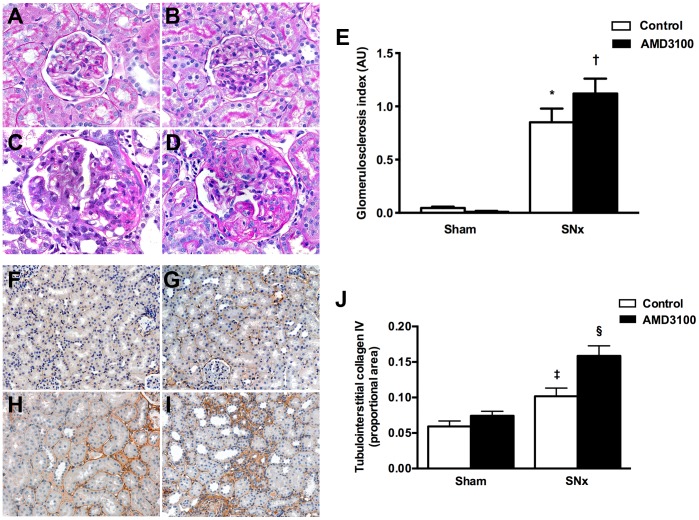
Effect of the CXCR4 antagonist, AMD3100 on glomerulosclerosis and renal fibrosis in subtotally nephrectomized (SNx) rats. PAS-stained kidney sections from sham (A and B) and SNx (C and D) rats treated with vehicle (A and C) or AMD3100 (B and D) for eight weeks. Original magnification x400. (E) Glomerulosclerosis index. Tubulointerstitial collagen IV immunostaining of kidney sections from sham (F and G) and SNx (H and I) rats treated with vehicle (F and H) or AMD3100 (G and I) for eight weeks. Original magnification x160. (J) Quantitation of tubulointerstitial collagen IV. AU = arbitrary units. *p<0.001 vs. sham, ^†^p<0.05 vs. SNx + vehicle, ^‡^p<0.05 vs. sham, ^§^p<0.01 vs. SNx + vehicle.

Having identified a dysregulation in SDF-1/CXCR4 expression in the kidneys of SNx rats, we next sought to determine the role of this pathway in the chronically ischemic kidney. Sham and SNx rats were therefore randomized to receive either vehicle (PBS) or the CXCR4 antagonist AMD3100 (1 mg/kg/day s.c.) for eight weeks. AMD3100 is a non-peptide, highly specific antagonist of CXCR4 (IC_50_ for calcium flux 572±190 nM vs. >100 μM for CCR1, CCR2b, CXCR3, CCR4, CCR5 and CCR7 [25]) that binds to the receptor through three primary acid residues Asp^171^ (AspIV:20), Asp^262^ (AspVI:23) and Glu^288^ (GluVII:06) in an irreversible or slowly reversible manner [26]. At the end of the eight week study period, systolic blood pressure (SBP) and urine protein excretion were increased while GFR was decreased in SNx rats relative to sham animals (Table 1). Change in each of these parameters was augmented in SNx rats receiving AMD3100, with a rise in SBP and urine protein and decrease in GFR relative to vehicle-treated SNx rats (Table 1). AMD3100 had no effect on SBP, urine protein excretion or GFR in sham rats (Table 1). Examination of kidney sections by light microscopy revealed that SNx surgery was associated with an expected increase in glomerulosclerosis (Figure 3A–E) and tubulointerstitial collagen IV deposition (Figure 3F–J), with both of these indicators of renal fibrosis being augmented with CXCR4 antagonism in SNx rats (Figure 3).

**Table 1:** Functional characteristics of sham and subtotal nephrectomy (SNx) rats treated with vehicle or AMD3100.

	Body weight (g)	Left kidney weight (g)	Left kidney weight / body weight (%)	SBP (mmHg)	GFR (ml/min/kg)	Urine protein excretion (mg/day)
Sham + vehicle	186±3	0.56±0.01	0.300±0.005	113±2	5.36±0.25	1.71×/÷1.12
Sham + AMD3100	185±5	0.54±0.02	0.296±0.005	112±3	5.29±0.28	2.37×/÷1.08
SNx + vehicle	172±3* ^†^	0.67±0.04^‡§^	0.400±0.031^§¶^	145±7^‡§^	3.22±0.32^‡§^	20.42×/÷1.52^‡§^
SNx + AMD3100	180±3	0.67±0.03^‡§^	0.378±0.016^†‡^	165±5^‡§||^	1.73±0.10^‡§^**	50.70×/÷1.26^‡§||^

SBP = systolic blood pressure.

*p<0.05 vs. sham + vehicle, ^†^p<0.05 vs. sham + AMD3100, ^‡^p<0.01 vs. sham + vehicle, ^§^p<0.01 vs. sham + AMD3100, ^¶^p<0.001 vs. sham + vehicle, ^||^p<0.05 vs. SNx + vehicle, **p<0.01 vs. SNx + vehicle.

### CXCR4 blockade accelerates capillary loss in subtotally nephrectomized rats

**Figure 4:**
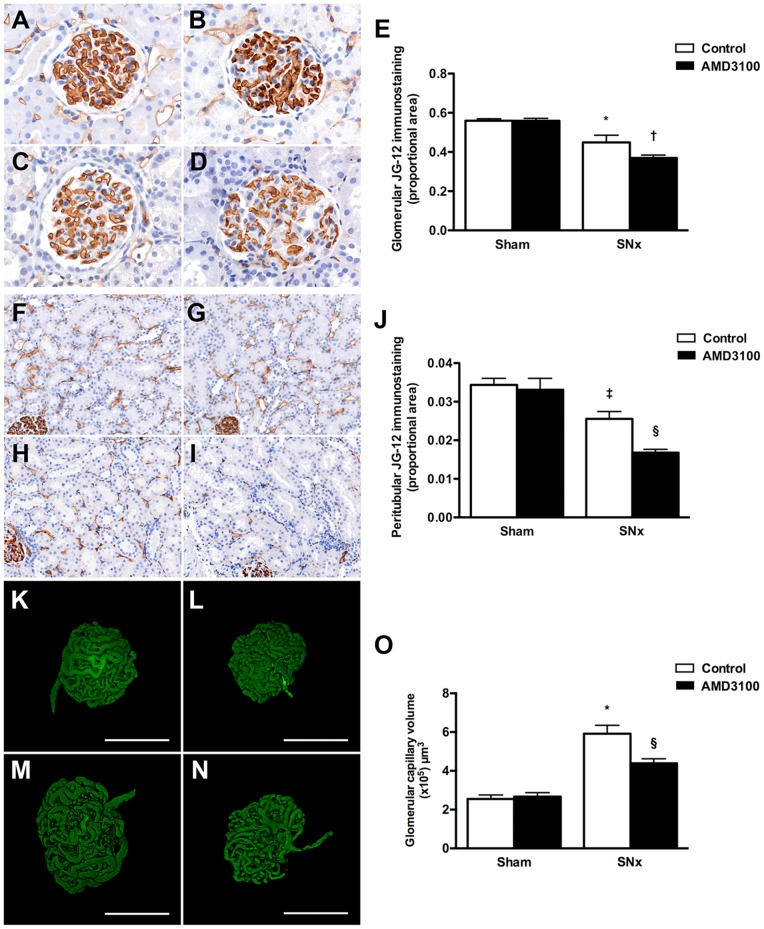
Effect of CXCR4 antagonism with AMD3100 on capillary loss in subtotally nephrectomized (SNx) rats. (A–D) Glomerular endothelial (JG-12) immunostaining of kidney sections from sham (A and B) and SNx (C and D) rats treated with vehicle (A and C) or AMD3100 (B and D) for eight weeks. Original magnification x400. (E) Quantitation of glomerular JG-12. (F–I) Peritubular JG-12 immunostaining of kidney sections from sham (F and G) and SNx (H and I) rats treated with vehicle (F and H) or AMD3100 (G and I). Original magnification x160. (J) Quantitation of peritubular JG-12. (K–N) Fluorescent microangiography (FMA) images of glomeruli from sham (K and L) and SNx (M and N) rats treated with vehicle (K and M) or AMD3100 (L and N) for eight weeks. (O) Glomerular capillary volume. *p<0.001 vs. sham, ^†^p<0.05 vs. SNx + vehicle, ^‡^p<0.01 vs. sham, ^§^p<0.01 vs. SNx + vehicle.

As CXCR4 was detectable in the endothelial cells of both the glomerular and peritubular capillaries we next sought to determine the effect of CXCR4 antagonism on both capillary density and capillary volume in sham and SNx rats. To assess the density of the glomerular and peritubular capillaries, kidney sections were immunostained with the monoclonal antibody JG-12 that binds to endothelial cells of capillaries but not lymphatics in rat kidneys [27]. Endothelial immunostaining was reduced in both the glomerular and peritubular compartments eight weeks after SNx surgery, with a further reduction in capillary density noted in kidney sections of AMD3100-treated SNx rats (Figure 4A–J). To determine whether glomerular capillary volume was also affected, we used the novel technique of fluorescent microangiography (FMA) that allows the generation of virtual three dimensional glomerular microvascular casts following confocal optical sectioning [18]. Consistent with the hypertrophic response that accompanies renal mass ablation, glomerular volume was increased in vehicle-treated SNx rats in comparison to sham rats and this was also significantly reduced with CXCR4 blockade (Figure 4K-O).

### Local SDF-1/CXCR4 signaling activates glomerular eNOS in the adult kidney

**Figure 5:**
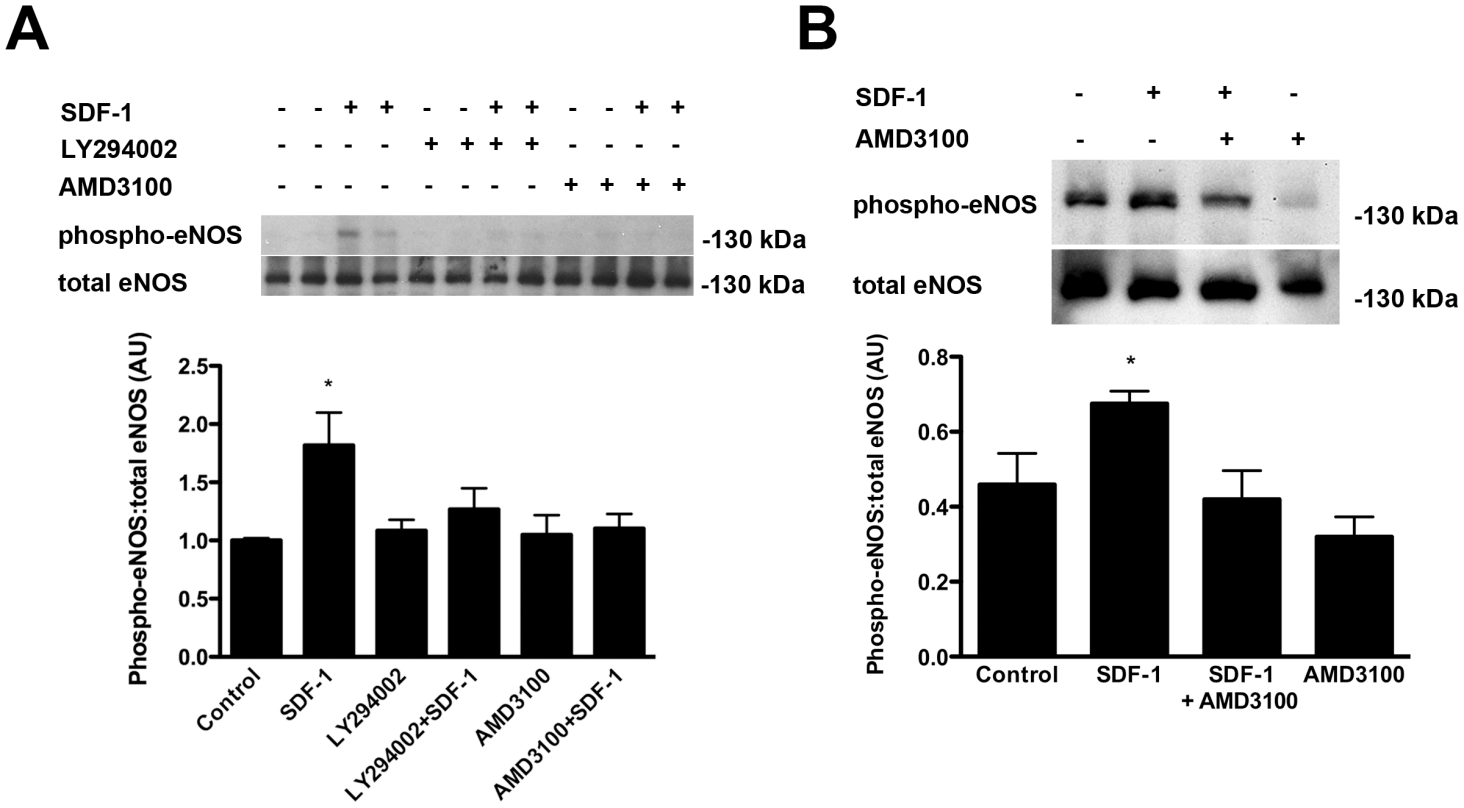
Phosphorylation of eNOS by SDF-1 at the activation site Ser1177. (A) Effect of SDF-1, the PI3-kinase inhibitor, LY294002 and the CXCR4 inhibitor, AMD3100 on eNOS Ser1177 phosphorylation in human umbilical vein endothelial cells (HUVECs). (B) Effect of acute SDF-1 administration on eNOS Ser1177 phosphorylation in sieved rat glomeruli following pre-treatment with PBS or the CXCR4 antagonist AMD3100. AU = arbitrary units. *p<0.05 vs. all other groups.

Since chronic CXCR4 antagonism accelerated renal decline and augmented capillary loss in SNx rats and since both the receptor and ligand were present in both the glomerular and tubulointerstitial compartments, we next sought to determine whether the SDF-1/CXCR4 axis may mediate local vascular signaling pathways in the adult kidney. One downstream regulator of the endothelial response to SDF-1, is endothelial nitric oxide synthase (eNOS) that may be activated by phosphorylation of its serine 1177 residue, through an Akt/phospho-inositide 3-kinase (PI3-kinase) dependent pathway [28]. To confirm the role of this pathway in endothelial eNOS activation, we first exposed cultured human umbilical vein endothelial cells (HUVECs) to recombinant SDF-1 with or without pre-treatment with either AMD3100 or the PI3-kinase antagonist, LY294002. Immunoblotting cell lysates confirmed that SDF-1 induced eNOS Ser1177 phosphorylation and that this was prevented by either CXCR4 or PI3-kinase inhibition (Figure 5A). To determine whether a similar signaling cascade is functional within the adult glomerulus, recombinant SDF-1 was next delivered to the kidneys of normal rats, via the abdominal aorta, with or without pre-treatment with AMD3100 (n = 6/group). Immunoblotting of glomeruli, isolated by differential sieving 30 min later, revealed that acute SDF-1 delivery induced an increase in eNOS Ser1177 phosphorylation and that this was antagonized by pre-treatment with AMD3100 (Figure 5B).

### SDF-1/CXCR4 expression is altered in the biopsies of patients with secondary focal segmental glomerulosclerosis

**Figure 6:**
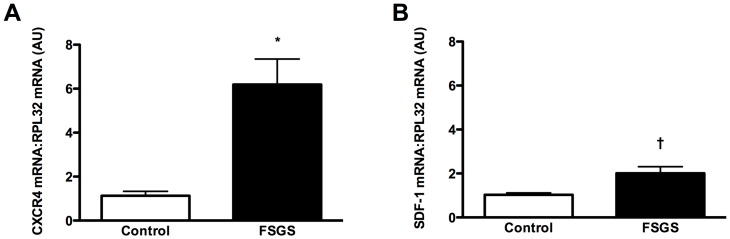
Real-time PCR for CXCR4 (A) and SDF-1 (B) mRNA in biopsies from time zero live kidney donors (Control, n = 10) and patients with secondary focal segmental glomerulosclerosis (FSGS, n = 6). AU = arbitrary units. *p<0.001, ^†^p<0.01.

Our experiments thus far had revealed that local SDF-1/CXCR4 signaling functions to preserve microvascular integrity and renal function in SNx rats, whereas the expression of both SDF-1 and CXCR4 is dysregulated following renal mass ablation, being characterized by downregulation of the former and upregulation of the latter. To determine whether the SDF-1/CXCR4 pathway is similarly dysregulated in human CKD, we examined gene expression of both receptor and ligand in biopsies from patients with secondary focal segmental glomerulosclerosis (FSGS) and time zero live kidney donor controls. FSGS is a common cause of progressive proteinuric kidney disease that bears pathophysiological similarity to the kidney injury that follows experimental renal mass ablation [29]. Sufficient archival formalin-fixed paraffin-embedded biopsy tissue was available to determine CXCR4 and SDF-1 expression in six samples from patients with biopsy-proven and clinically correlated obesity-related secondary FSGS compared with ten samples from time-zero live kidney donors. The clinical characteristics of patients with FSGS are shown in Table 2. All patients were hypertensive with an elevated urine protein excretion and two subjects had concomitant diabetes mellitus. For kidney donors, all patients were normotensive, with normal renal function and none of the donors had diabetes. In these studies, CXCR4 mRNA was observed to be increased approximately 5-fold in the kidneys of patients with FSGS relative to controls (Figure 6A), while there was a doubling in SDF-1 mRNA (Figure 6B).

**Table 2:** Clinical characteristics of patients with secondary focal segmental glomerulosclerosis (FSGS).

Patient	Age (years)	Weight (kg)	Sex (M/F)	Hypertension (Y/N)	ACEi/ARB	Diabetes (Y/N)	Urine protein excretion	Serum creatinine (μmol/L)
1	13	102.6	M	Y	Enalapril 10 mg o.d.	N	Protein:creatinine ratio 400 mg/mmol	83
2	34	102.6	M	Y	Ramipril 10 mg o.d.	N	24 h urine protein excretion 1.87 g/24 h	300
3	55	106.6	M	Y	Ramipril 10 mg o.d.	Y	Protein:creatinine ratio 679 mg/mmol	108
4	34	104.5	F	Y	No	N	Protein:creatinine ratio 338mg/mmol	76
5	17	154.9	M	Y	Enalapril 10mg o.d.	Y	Protein:creatinine ratio 118mg/mmol	53
6	38	116	M	Y	Enalapril 20mg o.d.	N	Protein:creatinine ratio 120mg/mmol	98

### ACE inhibition augments renal SDF-1 expression in CKD

**Figure 7:**
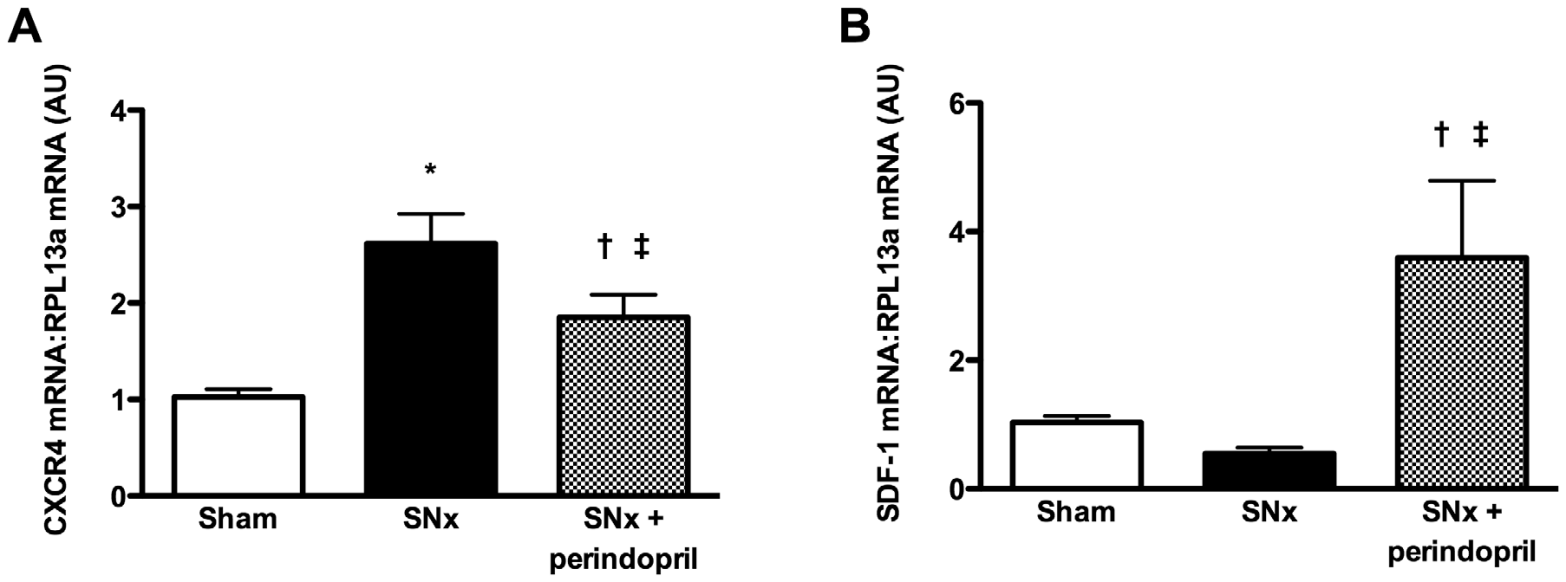
Real-time PCR for CXCR4 (A) and SDF-1 (B) mRNA in kidneys from sham rats, subtotally nephrectomized (SNx) rats and SNx rats treated with perindopril. *p<0.001 vs. sham, ^†^p<0.05 vs. sham, ^‡^p<0.05 vs. SNx.

Although CXCR4 expression was consistently increased in both experimental and human CKD, SDF-1 mRNA was more variable, with a decrease in expression in SNx kidneys and an increase in FSGS biopsies. In considering potential mechanisms that may contribute to this apparent discordance, we recognized that five of the six FSGS patients were treated with ACE inhibitors (Table 2). To determine whether either SDF-1 or CXCR4 expression may be altered by ACE inhibition, we finally examined mRNA levels in a separate cohort of rats that had been treated with the ACE inhibitor perindopril, as previously described [21]. These experiments revealed that CXCR4 mRNA was increased in SNx rats and, while reduced with perindopril-treatment, remained significantly elevated relative to sham rats (Figure 7A). Moreover, SDF-1 mRNA was markedly increased in the kidneys of perindopril-treated SNx rats when compared with either sham animals or SNx rats treated with vehicle, consistent with the change in gene expression observed in human FSGS biopsies (Figure 7B).

## Discussion

Despite existing therapies and regardless of the underlying etiology, renal function continues to decline in the majority of people with CKD. Over recent years, the almost universal failure of novel anti-albuminuric therapies to meaningfully impact on clinical outcomes has encouraged investigators to direct their attention to pathogenetic processes that commonly occur during the later stages of CKD development. These pathogenetic processes include renal fibrosis and the associated disruption in the microvascular architecture, acting together and compounding the deleterious effects of one another in the relentless progression towards end-stage renal disease. In the present study, we identified a novel role for SDF-1/CXCR4 signaling in this reciprocal relationship, observing that local SDF-1/CXCR4 signaling preserves microvascular integrity and attenuates fibrogenesis, whereas the pro-fibrotic growth factor TGF-β, overelaborated in the CKD setting, downregulates SDF-1 expression. Augmentation of SDF-1/CXCR4 signaling by novel or existing agents, either purposefully or serendipitously, may thus slow the progression of renal decline in CKD.

SDF-1 signaling through CXCR4 promotes cell survival [30], migration [28], and proliferation [31], favoring neo-angiogenesis both through direct effects and through the creation of a permissive microenvironment that facilitates the actions of the angiogenic factor, vascular endothelial growth factor (VEGF) [32], [33]. In the present study we observed that i) CXCR4 is expressed on the surface of endothelial cells in both the glomerular and peritubular compartments, ii) chronic CXCR4 blockade accelerates capillary loss in rats with CKD and iii) local SDF-1 delivery induces glomerular eNOS activation in a CXCR4-dependent manner. Moreover, accelerated disruption of the microvascular architecture with chronic CXCR4 blockade was associated with augmented renal decline and progressive renal fibrosis, analogous to the exacerbation of cardiac dysfunction with chronic CXCR4 blockade in the post-myocardial infarction setting [34]. Collectively, these observations highlight a hitherto unrecognized role for local SDF-1/CXCR4 signaling in preserving microvascular integrity and preventing coincident renal fibrosis in CKD.

In light of the detrimental effects observed with chronic CXCR4 blockade, the consistent upregulation of CXCR4 in the kidneys of SNx rats and in biopsies from patients with secondary FSGS is likely indicative of a compensatory response. CXCR4 upregulation has previously been described as a feature of a hypoxia-related glomerulopathy in patients with hypertensive nephrosclerosis [35]. Although both CXCR4 and SDF-1 are recognized as being hypoxia-responsive genes [36], their expression patterns were not indistinguishable in rat and human CKD. Whereas CXCR4 was consistently upregulated in both rats and humans with CKD, SDF-1 expression was more variable, the chemokine being notably downregulated in SNx kidneys of both female and male rats. In considering plausible mediators for SDF-1 downregulation in rats with CKD, we recognized the reciprocal relationship between fibrogenesis and capillary loss and hypothesized that decreased SDF-1 expression may occur as a consequence of the fibrotic process itself. TGF-β is a pro-sclerotic cytokine implicated in the fibrogenic response of many organs, including the kidney. Consistent with previous studies in oral myofibroblasts [37] and mimicking the changes in gene expression observed in the kidneys of SNx rats, exposure of cultured renal fibroblasts to recombinant TGF-β resulted in a marked downregulation in SDF-1 expression.

The post-translational modification of proteins, by the addition or removal of functional groups, is a common mechanism for controlling protein behaviour, perhaps most readily appreciated when considering the essential role that (de)phosphorylation plays in cellular homeostasis. More recently, post-translational protein modification through the addition or removal of acetyl groups has been recognized to rival phosphorylation in its diversity of substrates and the functional pathways affected [38]. (De)acetylation of proteins on lysine residues is regulated by the opposing actions of groups of enzymes called histone acetyltransferases and histone deacetylases (HDACs). In recent times, increasing evidence has begun to emerge for a pivotal role for HDACs in mediating both the progression of renal fibrosis and the response to TGF-β itself. For instance, work from our own group showed that HDAC inhibition attenuated glomerular matrix accumulation in diabetic mice [23], whereas acetylation may alter the cellular response to TGF-β through affecting Smad 2/3 activity [39], Smad7 stability [39] and/or the actions of downstream transcription factors [40] among other processes. Confirming that TGF-β mediated SDF-1 downregulation is under the regulatory control of protein acetylation, the decrement in SDF-1 transcript levels induced by TGF-β was attenuated by pre-treatment of cells with the HDAC inhibitor, vorinostat.

One of the challenges restricting the translation of promising experimental observations to the clinic is the limited ability of rodent models to recapitulate human disease. For instance, we observed that whereas SDF-1 expression was downregulated in SNx rats, the opposite effect occurred in biopsies from patients with secondary FSGS. This superficial discordance encouraged us to consider the differences between the human disease and experimental model. To account for the confounding effects of medication usage among patients, we therefore examined SDF-1 expression patterns in historical samples from SNx rats that had or had not been treated with the ACE inhibitor, perindopril [21]. In contrast to vehicle treatment, ACE inhibition, previously shown to decrease the overelaboration of TGF-β in SNx rats [41], resulted in a marked upregulation in SDF-1. Augmentation of SDF-1 activity may thus represent a novel mechanism by which renin angiotensin system blockade preserves the renal vasculature [42].

Although SDF-1/CXCR4 interaction was originally considered a monogamous relationship, more recent evidence suggests that this is not the case. CXCR4 also acts as a receptor for the HIV envelope receptor glycoprotein gp120 [43] and for the small protein ubiquitin [44], while SDF-1 may also bind to CXCR7. The renal actions of CXCR7 are complex with reports that the receptor signals in “renal multipotent progenitors” [45] while also functioning as a scavenger protein for SDF-1 [46]. Similarly, under some alternative conditions renal SDF-1/CXCR4 may play a potentially detrimental role through promoting podocyte proliferation [14], inflammatory cell recruitment [47] or, in the case of Shiga-toxin induced injury, endothelial phenotypic switch [48]. Thus, as with other angiogenic mediators [4], [5], the role of renal SDF-1/CXCR4 is likely to be contextual, varying according to stage of development and the underlying injurious insult. The collective *in vitro*, acute and chronic *in vivo* and human correlative studies herein described indicate a reno-protective function for this pathway in CKD.

In summary, SDF-1/CXCR4 signaling is not only important for renal vascular development but the same system also plays a pivotal role in preserving microvascular integrity in CKD. Augmentation of this pathway by novel or existing therapies may attenuate renal fibrosis and slow the progression of renal decline in CKD.
